# Acquired craniomeningocele in an infant with craniosynostosis: a case report

**DOI:** 10.1186/1752-1947-4-104

**Published:** 2010-04-11

**Authors:** Mostafa El Khashab, Farideh Nejat, Shahrooz Yazdani, Nima Baradaran

**Affiliations:** 1 Department of Neurosurgery, Children's Hospital Medical Center, Medical Sciences, University of Tehran, Tehran, Iran; 2 Department of Neurosurgery, Hackensack University Medical Center, New Jersey, USA

## Abstract

**Introduction:**

Craniosynostosis can affect the skull in various ways. The most common forms are abnormal skull shape and beaten copper pattern, while Lückenschädel (or lacunar skull) is one of the least common forms.

**Case presentation:**

We report the case of a 3-month-old Caucasian boy with multiple suture craniosynostosis and with acquired craniomeningocele presenting as a bulging mass in the lateral occipital area.

**Conclusion:**

To the best of our knowledge, this is the first report of a patient with multiple suture craniosynostosis and acquired craniomeningocele.

## Introduction

The incidence of non-syndromic craniosynostosis is approximately 0.4 to 1 in 10,000 live births. A single suture is most commonly implicated. Syndromic craniosynostosis, with a more generalized pattern, is much less common [1]. The majority of patients with craniosynostosis have various skull alterations, which are severe in syndromic type and are mainly considered to have arisen from compensatory growth of the skull after stenosis of some sutures and high intracranial pressure. Skull deformities and beaten copper pattern are common findings in craniosynostosis. Lückenschädel (lacunar skull) is rarely observed, occuring in approximately 10% of patients with craniosynostosis [2,3]. To the best of our knowledge, cranial meningocele has not been reported with craniosynostosis before. We describe a patient with craniosynostosis with acquired craniomeningocele and hypothesize probable causes for this abnormal presentation.

## Case presentation

**Figure 1:**
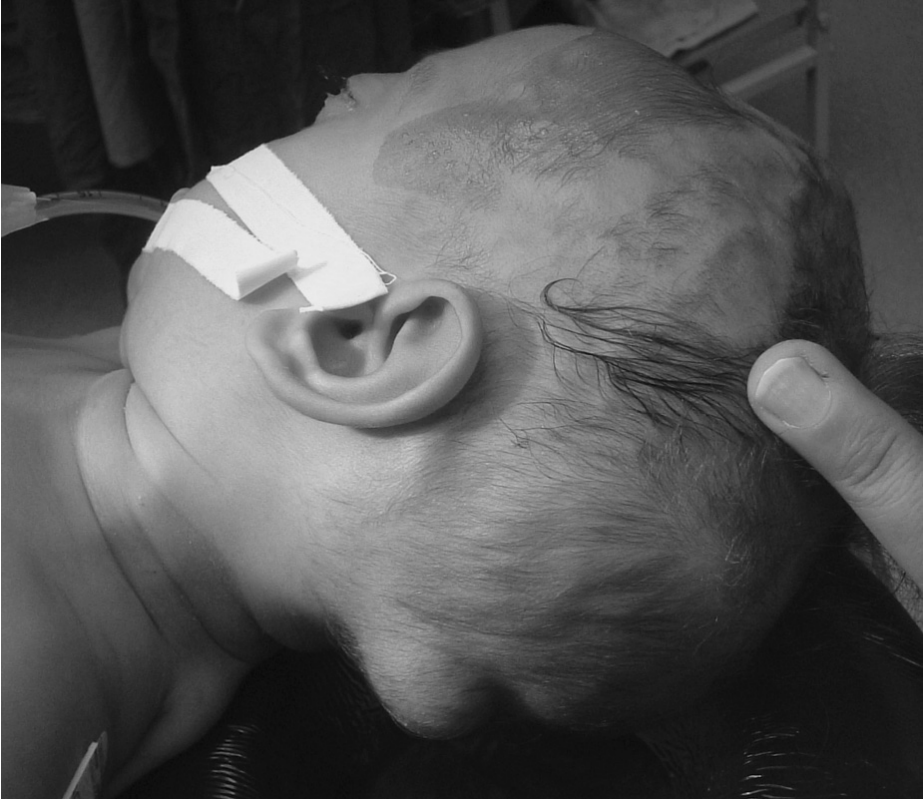
**Occipital lump: a unilateral mass in the left occipital area with pulsation but without tenderness**.

**Figure 2:**
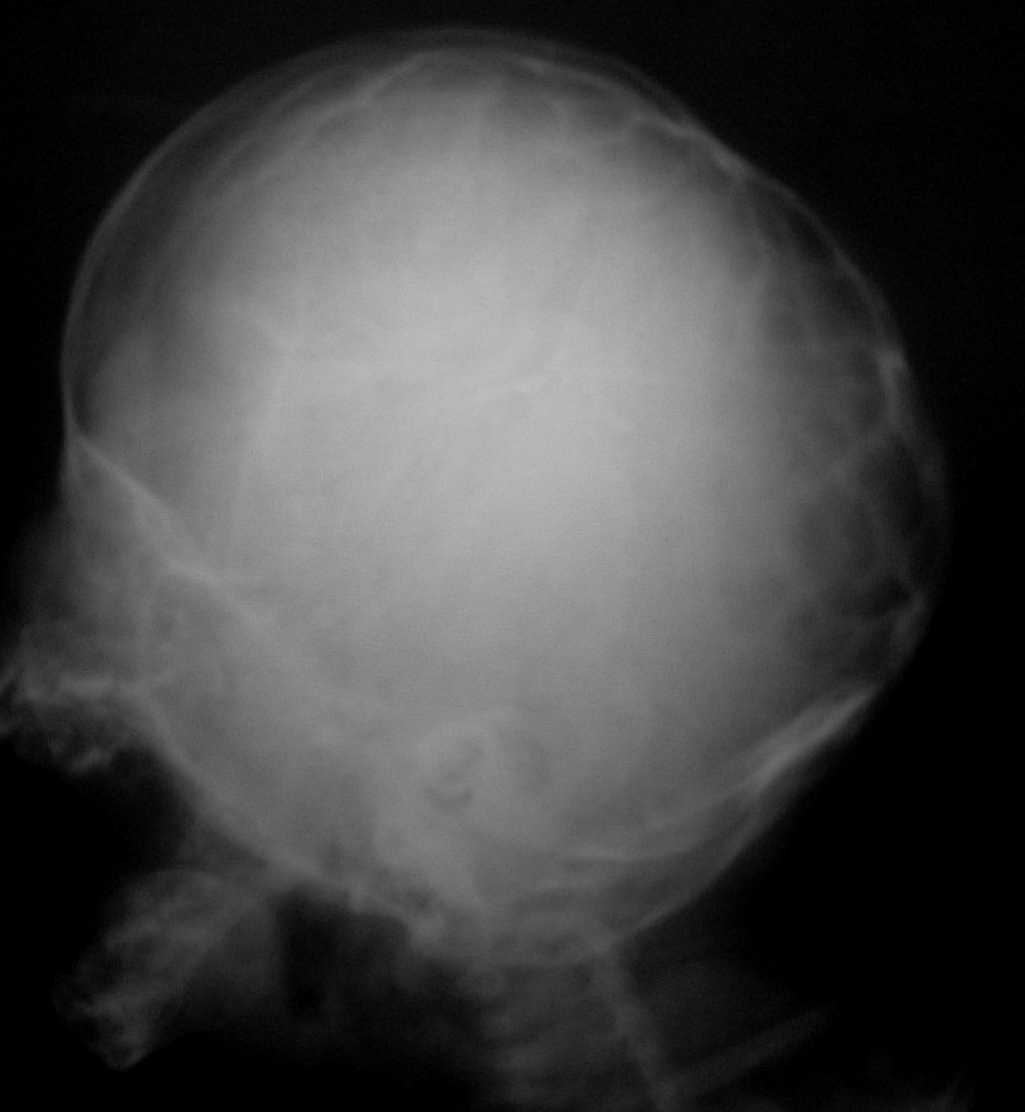
**Skull X-ray shows fusion of both coronal and lambdoid sutures with diffuse beaten copper appearance**.

**Figure 3:**
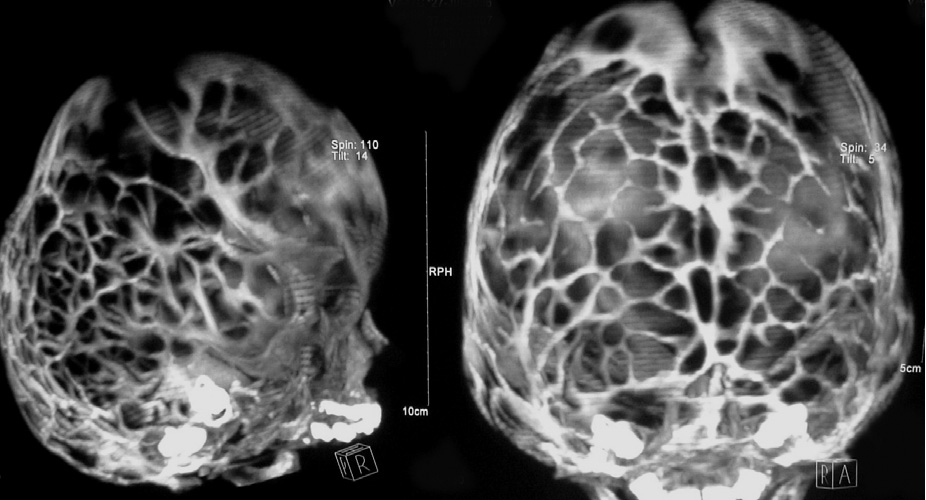
**Tri-dimensional computed tomography scan confirmed the fusion in the coronal and lambdoid sutures and diffuse skull changes with honeycomb configuration and beaten copper appearance**.

A 3-month-old Caucasian boy was referred to our hospital due to abnormal shape of his head and a bulging mass in the left occipital area. The mass had been found one month before referral with a gradual increase in size, and fluctuation in size during crying and sleeping. The lesion was 1.5 × 2 cm in maximum diameter (Figure 1), very tense, non-tender, and pulsatile. His head circumference was on the 25th percentile for his age class. There was apparent hypertelorism with exophthalmia and age-appropriate developmental milestones. Askull X-ray (Figure 2) and a brain computed tomography (CT) scan showed fusion of coronal, lambdoid and basal sutures with a diffuse beaten copper appearance (Figure 3). Hydrocephalus was not found in the CT scan. Expansive craniotomy associated with skull reconstruction was performed. There were prominent convolutional markings in the skull exposed in the operation field, complicated by dura matter entrapment in some places. The bone was very thin especially in posterior regions in conjunction with small defects in some areas. Postoperative course was uneventful and the child's occipital mass resolved dramatically.

## Discussion

Skull shape irregularity has been recognized since antiquity, but the scientific study of abnormal skull growth related to craniosynostosis originates back to the late 1700s. Experimental studies have shown that selective restriction of an individual cranial vault suture's growth could result in skull deformities [4]. Moreover, cranial base and even facial deformities develop secondary to the cranial vault suture restrictions [5].

Beaten copper pattern is another skull change observed in craniosynostosis. It was initially assumed to have resulted from chronically elevated intracranial pressure (ICP) [6], but later it was widely considered to be a reflection of normal brain growth, without pathological significance [7]. Diffuse, severe beaten copper pattern is an indicator of elevated ICP, which is more common in patients with craniosynostosis than in unaffected children. The beaten copper pattern originates from the pressure that the brain exerts on the continuously remodeling inner cranium resulting in bony convolutions that are manifested as a beaten copper appearance on cranial radiographs [8].

Lückenschädel is characterized by multiple, round or oval, radiolucent defects, sharply separated by dense strips of bone (honeycomb-like configuration), which tend to cluster in the cranial vault on plain skull film. It can be a consequence of elevated ICP or can be a self-limited phenomenon seen in children with myelomeningocele [9].

A comprehensive Medline search of articles from 1966 to 2008 revealed no previous cases of acquired craniocele in craniosynostosis. The development of craniocele in the setting of multiple suture synostosis is not clearly understood and some suggestions may be considered. Severe synostosis and reduced cranial growth in this patient caused brain volume to exceed the cranium size, resulting in severely elevated ICP. High ICP was observed in a brain CT scan as multiple sites of bone defects, diffuse beaten copper appearance and Lückenschädel. Meanwhile in the occipital area, where the cranium and brain are closely associated, high ICP produced a gross bone defect and dura with brain herniated through this defect to attenuate the pressure.

## Conclusion

The presence of brain herniation in a patient with multiple suture synostosis is not clearly understood and has not been reported before. Our patient presented with occipital craniocele and we have tried to explain the probable mechanisms causing this lesion. Severe synostosis coupled with reduced cranial growth caused severe intracranial hypertension. Meanwhile in the occipital area, where the cranium and brain are closely associated, high ICP can result in a bone defect and dura associated with brain herniated through this defect.

## Abbreviations

CT: computed tomography; ICP: intracranial pressure.

## Consent

Written informed consent was obtained from the patient's parents for publication of this case report and any accompanying images. A copy of the written consent is available for review by the Editor-in-Chief of this journal.

## Competing interests

The authors declare that they have no competing interests.

## Authors' contributions

FN, NB, and SY all made major contributions in patient care, literature review and drafting of the manuscript. MEK made a substantial contribution to the literature review, correction and final approval of the manuscript. All authors read and approved the final manuscript.
